# The Changing Landscape of Lymphoma Associated with HIV Infection

**DOI:** 10.1007/s11912-020-00973-0

**Published:** 2020-08-15

**Authors:** Kai Hübel

**Affiliations:** Faculty of Medicine and University Hospital of Cologne, Department I of Internal Medicine, University of Cologne, Kerpener Str. 62, 50937 Cologne, Germany

**Keywords:** HIV lymphoma, Pathogenesis, Treatment, Risk factors, Antiretroviral therapy

## Abstract

**Purpose of Review:**

Cancer remains a major cause of morbidity and mortality in HIV-infected individuals, with aggressive non-Hodgkin’s lymphoma as the most frequent one. However, the introduction of modern antiretroviral therapy (ART) drastically improved treatment options and prognosis in HIV-associated lymphomas. This review summarized the current treatment landscape and future challenges in HIV-positive patients with non-Hodgkin’s and Hodgkin’s lymphoma.

**Recent Findings:**

Selecting the appropriate therapy for the individual patient, diffuse-large B cell lymphoma, Burkitt’s lymphoma, and Hodgkin’s disease may be curable diseases. In contrast, the prognosis of plasmablastic lymphoma and primary effusion lymphoma remain poor. New treatment approaches, as targeted therapies or CAR T cell therapy, may broaden the therapeutic armamentarium.

**Summary:**

The continuous application of ART is mandatory for successful treatment. The choice of lymphoma therapy may follow the recommendations for HIV-negative patients, but prospective trials in HIV-lymphoma are needed.

## Introduction

At the beginning of the 1980s, morbidity and mortality of the acquired immune deficiency syndrome (AIDS) were mainly associated with opportunistic infections such as *Pneumocystis jirovecii* (formerly known as *P. carinii*) or infections with cytomegalovirus (CMV). However, it was quickly learned that patients infected with the human immunodeficiency virus (HIV) also have a high risk of developing several forms of cancer. These malignancies may arise when the CD4 T cell count is low and the immune system is compromised. Virally induced neoplasia such as Kaposi’s sarcoma, cervical cancer, and aggressive non-Hodgkin’s lymphoma (NHL) have been defined as AIDS-related cancers. Nowadays, this definition is somewhat anecdotal. With the introduction of combination antiretroviral therapy (cART) in 1996, not only the risk of opportunistic infections declined but also the risk of developing cancer. Furthermore, the availability of cART broadens the spectrum of therapeutic options for the treating oncologist.

Nevertheless, cancer remains a major cause of mortality in HIV-infected individuals, with HIV-associated NHL as the most frequent one [1]. Being infected with HIV is associated with an increased risk for the development of lymphoid malignancies as compared with the general population. The most frequent subtypes of HIV-associated NHL are the diffuse large B cell lymphoma (DLBCL) and the Burkitt’s lymphoma (BL) [2]. The classical Hodgkin’s lymphoma (HL) is one of the most common non-AIDS defining malignancies, with a 5- to 20-fold higher risk compared with HIV-negative individuals [3]. There is no doubt that understanding of the underlying mechanisms of HIV-associated lymphoma development and optimization of treatment strategies will significantly help to further decrease morbidity and mortality of HIV-infected people.

### Pathology and Pathogenesis

The World Health Organization classification 2017 divided HIV-associated lymphomas in three groups [4] as follows: (1) lymphoma also occurring in immunocompetent patients. These are primarily the DLBCL, the BL, and the HL. (2) Lymphomas occurring more specifically in HIV-positive patients. These are primarily the primary effusion lymphoma (PEL) and the plasmablastic lymphoma (PBL). (3) Lymphomas occurring in other immunodeficient states. This category includes post-transplant lymphoproliferative disorders and accounts for less than 5% of HIV lymphomas.

In general, lymphomas in HIV-infected patients are heterogenous, reflecting a variety of pathogenetic mechanisms as follows: chronic antigen stimulation, genetic abnormalities, cytokine deregulation, and the role of different viruses [4]. Epstein Barr virus (EBV) is identified in about 40% of the neoplastic cells of HIV-related lymphomas. PEL is often associated with human herpes virus 8 (HHV8) infection [5]. Furthermore, the degree of CD4 cell depletion has a major impact on the type of lymphoma that develops. In patients with low CD4 cell count (< 200/μl), the incidence of lymphoma subtypes as immunoblastic DLBCL, PEL, or PBL increases. In patients with higher number of CD4 cells, centroblastic DLBCL and BL are more likely to occur [6].

There are more changes which may promote B cell dysregulation and therefore precede the development of HIV-associated lymphoma. Interleukins such as IL-10 and IL-6 and also levels of serum free light chains are found to be elevated prior to the diagnosis of HIV lymphoma [7, 8]. It is unclear if these changes are really predictive, and therefore, they are not used for routine screening of asymptomatic HIV patients.

### Risk Factors

There is strong evidence that HIV may directly drive lymphomagenesis but also HIV viremia and depth of CD4 nadir increase lymphoma risk [9, 10]. Furthermore, delayed initiation or interruptions in cART application fostered lymphoma development [11]. Following diagnosis of HIV-related lymphoma, several risk factors have been identified which are associated with a higher risk of relapse.

Table 1 summarizes important risk factors for lymphomagenesis and lymphoma outcome in HIV-positive patients.

**Table 1:** Summary of risk factors contributing to HIV lymphomagenesis and prognosis

Risk factors for HIV-lymphomagenesis	
Continuous immunosuppression, low CD4 cell count Patient not receiving ART or interruptions of ART HIV viremia Coinfection with HHV8, EBV, hepatitis B or C Loss of EBV-specific immunity	
Risk factors for poor outcome of HIV-lymphoma	
Continuous low CD4 cell count Patient not receiving ART HIV viremia High-risk age-adjusted IPI Extranodal involvement Prior history of AIDS	

*AIDS* acquired immune deficiency syndrome, *ART* antiretroviral therapy, *EBV* Epstein Barr virus, *HHV* human herpes virus, *HIV* human immunodeficiency virus, *IPI* international prognostic index. *Adapted from* [12–14, 15•]

### Antiretroviral Therapy

In the pre-cART era, the NHL risk was 25- to 150-fold higher in HIV-infected patients than in the general population. There is no doubt that the availability of cART significantly improved the prognosis of patients with HIV lymphoma. For example, comparing the pre-cART era with the cART era the 2-year overall survival (OS) in patients with DLBCL increases from 24% to 67% [16]. In BL, 2-year OS increases from 37% to 75%. However, these impressive results have not been seen in all lymphoma subtypes. In a small cohort of patients with PEL, OS improves marginally from 33% to 40% between the two eras. [17].

A delayed introduction of cART has been shown to significantly increase the risk for developing HIV lymphoma [18]. Initiation of cART should be prompt, irrespective of the CD4 cell count and independently whether chemotherapy is planned or not. There is no reason to delay cART because of chemotherapy, including high-dose chemotherapy [19, 20•]. However, this does not mean that interactions could be ignored. Integrase inhibitors bear advantages concerning drug-drug interactions and confer a faster decline of the viremia. By contrast, CYP3A4-inhibitors such as ritonavir and cobicistat-containing regimens should be avoided wherever possible to prevent severe adverse effects [21, 22].

Important factors which may influence treatment of HIV-associated lymphomas are displayed in Table 2.

**Table 2:** Factors influencing therapy of HIV-associated lymphomas

Lymphoma-related	Patient-related
Histology Stage Size Localization IPI	Age ECOG Comorbidity Compliance
HIV-related	Oncologist-related
CD 4 cell count Virus load Infections ART	Experience in lymphoma therapy Experience in HIV therapy

*ART* antiretroviral therapy, *ECOG* Eastern cooperative Oncology Group, *HIV* human immunodeficiency syndrome, *IPI* international prognostic index

### Diagnosis and Staging

To determine the specific HIV lymphoma type, diagnosis should be based on a tissue biopsy if even possible and not just on a fine needle aspiration. Pretreatment evaluation did not differ from the recommendations for HIV-negative patients with malignant lymphomas, including medical history and comorbidities, physical examination, and diagnostic interventions such as bone marrow biopsy, CT scans, or echocardiography [23]. Due to a similar transmission route, HIV patients have an increased risk to be coinfected with hepatitis B and C. Therefore, testing for hepatitis is mandatory.

There is still an ongoing discussion on the specificity and sensitivity of positron emission tomography (PET) in HIV-related lymphoma. In the HIV-negative patients, PET is well established in DLBCL and HL, but in lymphomas associated with HIV, nodal reactive hyperplasia and infections may confound PET interpretations [24]. In a phase II trial in 45 patients with HIV-DLBCL, it was shown that PET has an excellent negative but poor positive predictive value [25]. The role of PET should be further evaluated in clinical trials, but at this time, it should be interpreted with caution in HIV-associated lymphomas.

### Diffuse Large B Cell Lymphoma

DLBCL represents the most frequent subtype of lymphoma irrespective of HIV status. However, in HIV-positive patients, DLBCL is more often associated with high-risk factors as *MYC* or *BCL6* translocations or proliferation indices > 80% [26, 27]. In general, therapeutic approaches follow the recommendations for HIV-negative patients. The most common chemotherapy in HIV-DLBCL is the CHOP-regimen, consisting of cyclophosphamide, doxorubicin, vincristine, and prednisone. Other frequently used regimens are CHOEP (CHOP plus Etoposide) or EPOCH, which consisted of a 96-h intravenous infusion of etoposide, doxorubicin, and vincristine plus oral prednisone followed by intravenous bolus of cyclophosphamide. These regimens are highly effective in DLBCL, even if the outcome is less than in HIV-negative patients.

The use of rituximab in HIV-associated lymphomas is still discussed controversially, since it may influence control of HIV infection. However, there is no doubt that addition of rituximab to chemotherapy significantly improves the outcome. In a pooled analysis of 1546 patients with aggressive HIV-associated NHL (84% of them with DLBCL), rituximab plus chemotherapy was associated with a higher rate of complete remission (CR) (odds ratio 2.89; *p* < 0.001), improved progression-free survival (PFS) (hazard ratio (HR) 0.50; *p* < 0.001), and overall survival (OS) (HR 0.51; *p* < 0.0001) [12]. However, this benefit was not shown if CD4 count was below 50 cells/μl. In an analysis of the German HIV lymphoma cohort, 163 patients with aggressive HIV lymphomas were treated with (*n* = 71) or without (*n* = 58) rituximab [28]. It was clearly shown that the use of rituximab was associated with better OS and PFS (HR 0.48, 95% CI 0.25–0.93 and HR 0.47, 95% CI 0.26–0.86). In this trial, it was also shown that rituximab is also effective in severely immunosuppressed patients (CD4 count below 100 cells/μl) and not associated with a higher risk of fatal infections. In contrast, the AIDS malignancy consortium demonstrated in a randomized study a significant better CR rate if rituximab was added to chemotherapy but no benefit in PFS or OS [29]. These results were at least partially abrogated by an increased death rate due to infectious complications, particular in patients with a CD4 count below 50 cells/μl. Thus, the use of rituximab could be clearly recommended if the patient has a CD4 cell count > 50 cells/μl, but has to be given with caution in patients with very low CD4 counts.

The EPOCH regimen is still often used in HIV-DLBCL with documented efficacy. In a phase II trial with a short-course EPOCH regime combined with dose-dense rituximab (SC-EPOCH-RR), after a 5-year follow-up, the PFS was 84% and the OS was 68%, respectively [25]. However, in a retrospective comparison with R-CHOP, R-EPOCH failed to demonstrate a significant benefit in OS [12]. The equal efficacy of R-EPOCH and R-CHOP has also been shown in immunocompetent patients [30••].

Table 3 provides an overview about prospective clinical trials in aggressive HIV-associated lymphomas.

**Table 3:** Summary of frontline prospective trials in HIV-associated aggressive lymphomas

Regimen	*n*	Trial design	Subtype	Follow-up	CR	PFS/FFS	OS	Reference
EPOCH	39	Phase II	DLBCL 79% BL 18%	53 months	74%	92%	60%	[31]
CDE	55	Phase II	DLBCL 78% BL 22%	24 months	45%	38%	45%	[32]
CHOP	72	Phase II	DLBCL 61% BL 11% HG NOS: 24%	47 months	63%	N/A	26,1 months	[33]
CHOP	50	Phase III	DLBCL 80% BL 9% HG NOS 6%	137 weeks	47%	38 weeks	110 weeks	[29]
R-CHOP	99				58%	45 weeks	139 weeks	
R-CDE	74	Pooled analysis, phase II	DLBCL 72% BL 28%	23 months	70%	59%	64%	[34]
R-CHOP	61	Phase II	DLBCL 71% BL 29%	33 months	77%	69%	75%	[35]
R-CHOP	81	Phase II	DLBCL 100%	3 years	69%	77%	56%	[36]
R-EPOCH	110	Phase II, randomized	Arm A R-concurrent DLBCL 69% BL 31% Arm B R-sequential DLBCL 80% BL 20%	30 months	73% 55%	66% 62%	70% 67%	[37]

*BL* Burkitt’s lymphoma, *CDE* cyclophosphamide-doxorubicin-etoposide, *CHOP* cyclophosphamide-doxorubicin-vincristine-prednisolone, *CR* complete remission, *DLBCL* diffuse-large B cell lymphoma, *EPOCH* etoposide-prednisolone-vincristine-cyclophosphamide-doxorubicin, *FFS* failure-free survival, *HG NOS* high grade not otherwise specified, *N/A* not available, *OS* overall survival, *PFS* progression-free survival, *R* rituximab

At this time, it is still unclear if the cell of origin is prognostic in HIV-DLBCL. In the general population, the germinal center B cell-like (GCB)–subtype is associated with a more favorable prognosis compared to non-GCB lymphomas [38]. This was also shown in a phase II study in 33 patients with HIV-DLBCL who received three to six cycles SC-EPOCH-RR [25]. Five-year PFS was reported as 95% in GCB-subtype compared with 44% in non-GCB-subtype. In contrast, a retrospective analysis of six cycles of R-EPOCH or R-CHOP/CHOP in 81 patients showed the same survival in GCB and non-GCB HIV-DLBCL [39].

Data on the use of central nervous system (CNS) prophylaxis in HIV-DLBCL are rare and not based on controlled studies. CNS diagnostic intervention and CNS prophylaxis should be used according to the recommendations for non-HIV DLBCL [40]. Intrathecal or systemic strategies for CNS prophylaxis are used, but those patients identified with high risk of CNS relapse, intravenous high-dose methotrexate (MTX) may be more effective than intrathecal MTX [41].

### Burkitt’s Lymphoma

BL accounts to the most aggressive lymphoma at all, with proliferation indices of 95% or higher [42]. Therefore, durable remission or even cure requires intensive chemotherapeutic approaches. Due to molecular similarities between BL and B cell acute lymphoblastic leukemia, the majority of patients receive leukemia-based regimens. Frequently used therapy regimens are CODOX-M/IVAC (cyclophosphamide, doxorubicin, vincristine, methotrexate, etoposide, ifosfamide, cytarabine) or HyperCVAD/HD-MTX (hyperfractionated cyclophosphamide, vincristine, doxorubicin, and dexamethason plus high-dose methotrexate). In combination with rituximab, these regimens demonstrated CR rates between 63 and 93% [43–45]. Reported toxicity was in the range of experiencers in HIV-negative patients. Another well-known regimen is the B-ALL/NHL protocol of the German Multicenter Study Group for the Treatment of Adult ALL (GMALL), combining short-intensive cycles of chemotherapy and rituximab [46]. In a comprehensive analysis of two cohorts, 81 patients with HIV-related BL received the B-ALL-protocol, with dose reductions for patients older than 55 years [47]. This regimen was highly effective with a CR-rate of 80%. After a median follow-up of 48 months, PFS was 71% and OS was 72%. However, compared with the HIV-negative population, 11% of patients died due to treatment-related side effects.

Promising approaches have been reported with R-EPOCH-based regimens; however, the number of patients included in these trials is rather small. In 11 patients with HIV-BL, SC-EPOCH-RR achieved a PFS of 100% and an OS of 90% after a median follow-up of 72 months [48]. No patient died because of treatment-related toxicities. Similar results have been shown in 113 patients (29 of them with HIV-BL) receiving a dose-adjusted R-EPOCH [49•]. After a short median follow-up of 10 months, PFS was 86% and OS was 86%. Especially patients without CNS- or bone marrow involvement had an excellent outcome. The rate of treatment-related deaths was 4.6%. However, the number of patients in these trials is small. Further studies are needed before valuable recommendations of R-EPOCH-based regimens in HIV-BL can be made.

### Plasmablastic Lymphoma

PBL has initially been described in the pre-ART era as being an own entity and nearly exclusively associated with HIV [50]. Nowadays it is considered as a rare variant of DLBCL, with loss of CD20 and expression of myeloma markers such as CD38 and CD138. Patients often present with stage I jaw mass, but also disseminated disease at time of diagnosis is possible. Since PBL accounts only for 3–12% of all HIV-related lymphomas, data from clinical studies are very limited. Chemotherapy with CHOP or CHOP-like regimens demonstrated poor outcome. Also, more intensive approaches such as the B-ALL/NHL protocol, or high-dose chemotherapy followed by hematopoietic stem cell transplantation, were disappointing. The median OS was 11 months regardless of intensity of chemotherapy applied [51]. In a comprehensive analysis of 135 patients, HIV-positive patients had a better outcome compared with HIV-negative patients [52•]. There are some very limited reports using compounds with well-known activity in multiple myeloma patients. Castillo et al. combine bortezomib with EPOCH in three patients with PBL; two of them were HIV positive [53]. These two patients achieved an OS of 18 months and 24 months. Very recently, Ando et al. presented a case of a 64-year-old man with HIV-negative PBL refractory to conventional chemotherapy [54]. This patient responded to CyBorD (bortezomib, cyclophosphamide, dexamethasone), followed by lenalidomide/dexamethasone with a partial remission over 2 years. New approaches include EBV-directed therapies or EBV-targeted cellular immunotherapy with no clinical data yet [55]. Another trial combining daratumumab with DA-EPOCH in HIV-PBL is currently enrolling patients [56].

### Primary Effusion Lymphoma

PEL is another rare disease with no established therapy and poor outcome. It accounts for 1–5% of HIV-related lymphoma cases and expresses an immunoblastic or plasmablastic morphology, with loss of CD20 but expression of CD45, CD30, CD38, and CD138 [57]. All cases are associated with HHV 8 infection, and most cases also show coinfection with EBV [58]. Furthermore, PEL may extend into tissues underlying the pleural, pericardial, and peritoneal cavities. In an analysis of four clinical studies including 7–28 patients with PEL, 72–100% of patients received a CHOP or CHOP-like chemotherapy [59]. The CR rate was 43–57% with a median survival time between 5 and 9 months. In a large single-center series, 51 patients with PEL in the era of cART, median OS was 10.2 months with no difference between the cavitary or extracavitary groups [60]. Novel treatment approaches such as bortezomib, lenalidomide, or the use of antiretroviral drugs such as cidofovir or valganciclovir are currently a matter of ongoing research [61, 62].

### Primary Central Nervous System Lymphoma

HIV-related primary CNS-lymphoma is a rare disease and typically affects patients with CD4 counts < 50 cells/μl. The prognosis significantly improved with the availability of cART allowing intensive chemotherapy including hematopoietic stem cell transplantation. In a retrospective analysis of 57 patients of the pre-cART era receiving whole brain radiotherapy, median OS was 2.5 months, whereas in 20 patients of the cART era who received a high-dose methotrexate-based therapy, median OS was not reached with a median follow-up of 27 months [63]. In an analysis of 20,677 persons in the Center for AIDS Research Network of Integrated Clinical Systems cohort, the 2-year mortality rate of AIDS-related CNS lymphoma was 90.6% per 100 person-years, despite the availability of cART [64]. Nowadays, treatment of choice is high-dose chemotherapy including methotrexate and cytarabine, followed by autologous stem cell transplantation for responding patients. Concomitant cART is mandatory, and not receiving cART within 30 days of diagnosis is a risk factor for death [65].

### Hodgkin’s Lymphoma

Although HL associated with HIV is not an AIDS-defining disease, HIV infection increases the risk for developing HL [66]. Patients with HIV-HL frequently present with B symptoms or cytopenias, since bone marrow involvement is found in 40–50% of cases. HIV-HL is also often associated with EBV infection when compared with the HIV-negative population [13]. In a German prospective HIV cohort study, 86 patients with HL were compared with 329 patients with NHL [67]. Patients with HL were more likely to be on ART (73.5% vs. 39.1%, respectively; *p* < 0.001) and more frequently had a viral load below the detection limit (57.3% vs. 27.9%, respectively; *p* < 0.001) than patients with NHL. These data suggest that ART provides insufficient protection from developing HL. Interestingly, within the last and within the next-to-last year preceding HL diagnosis, mean CD4 T cells decreased by − 168 and by − 2 cells/μl and mean CD8 T cells decreased by − 352 and − 115 cells/μl [68].

A stage-adapted treatment approach is the current state of the art of therapy for patients with HL regardless of HIV status [69]. Patients with early favorable disease should receive two cycles of ABVD (adriamycin, bleomycin, vinblastine, dacarbazine) followed by 20 Gy involved-field radiotherapy whereas patients with early unfavorable disease should receive four cycles of ABVD plus subsequent 30 Gy involved-field radiotherapy. BEACOPP baseline (bleomycin, etoposide, adriamycin, cyclophosphamide, vincristine, procarbazine, prednisolone) or 6–8 cycles of ABVD is treatment of choice for patients with advanced stage. The latter is treatment of choice for patients with advanced HIV infection. Using this approach, the outcome of HIV-HL is quite good and may approach that of patients with HIV-negative HL. This was shown in a prospective multicenter trial in 108 patients with HIV-HL [70]. Twenty-one percent of these patients had early favorable HL, 13% had early unfavorable HL, and 66% had advanced-stage HL. The complete remission rates for patients with early favorable, early unfavorable, and advanced-stage HL were 96%, 100%, and 86%, respectively. The 2-year PFS of the entire study population was 91.7%; the 2-year OS was 90.7%.

Several new compounds such as immune checkpoint inhibitors or antibody drug conjugates opened new treatment options with favorable results in HIV-negative HL. In HIV-HL, there are only limited data on these approaches published so far. In a phase II trial, brentuximab vedotin was used in combination with AVD (sparing bleomycin) in stage II–IV HIV-HL [71]. Forty-one patients were enrolled, 83% of them with stage III/IV disease. The 2-year PFS was 86%; the 2-year was OS 92%. No new safety signals were recorded. These data document that brentuximab vedotin is also effective in HIV-HL.

### Relapsed Lymphoma

Regardless of the underlying type of lymphoma, there is no standard approach for relapsed or refractory HIV-associated lymphoma. In most cases, investigators will follow the recommendations for HIV-negative patients, combined with continuous cART application. However, one major question will be the use of high-dosed chemotherapy followed by hematopoietic stem cell transplantation in relapsed HIV lymphoma. In the largest series ever on lymphoma transplants in HIV-positive patients, the European Society for Blood and Marrow Transplantation (EBMT) recently analyzed 118 patients with various lymphoma subtypes undergoing autologous transplantation [20•]. With a median follow-up of 4 years, 3-year non-relapse mortality, incidence of relapse, PFS and OS were 10%, 27%, 63%, and 66%, respectively. By multivariate analysis, disease status less than PR but not CD4+ cell count at the time of autologous transplantation was a significant predictor of unfavorable PFS and OS. The authors concluded that autologous transplantation is safe and effective in patients with HIV-related lymphoma and should be considered according to the same criteria adopted for HIV-negative patients.

There are only limited data on the use of allogeneic transplantation in relapsed HIV-associated lymphoma, suggesting that this approach may be feasible [72, 73]. Chimeric antigen receptor (CAR) T cell therapy is a promising new class of cellular immunotherapy showing activity in several hematologic malignancies. These T cells are genetically modified to express CARs which recognize specific tumor targets and inducing an immune response leading to partial or complete tumor eradication [74]. In a small report of two patients with refractory HIV-associated high-grade B cell lymphoma, CAR T cells could induce a durable remission of even more than 1 year without influencing control of HIV infection [75].

## Conclusion

Overall, the outcome of patients with HIV-associated lymphoma significantly improved over the past decades, especially due to the availability of cART. The choice of treatment depends on patient’s performance status and active infections, but in most cases, therapy will follow the recommendations for HIV-negative patients. However, despite the use of intensive protocols in the cART era, the outcome of HIV lymphoma is still worse compared with the HIV-negative population. In the relapsed situation, autologous and allogeneic stem cell transplantation and even CAR T cell therapy may be feasible but will need further evaluation in prospective trials. Future directions include the delineation of the most effective but less toxic therapy but also the identification of new driver pathways and further therapeutic targets.
